# Impact of Gallic Acid on Gut Health: Focus on the Gut Microbiome, Immune Response, and Mechanisms of Action

**DOI:** 10.3389/fimmu.2020.580208

**Published:** 2020-09-16

**Authors:** Kang Yang, Limeng Zhang, Pinfeng Liao, Zaili Xiao, Fan Zhang, Daniel Sindaye, Zhongquan Xin, Chengquan Tan, Jinping Deng, Yulong Yin, Baichuan Deng

**Affiliations:** ^1^Maoming Branch, Guangdong Laboratory for Lingnan Modern Agriculture, Guangdong Provincial Key Laboratory of Animal Nutrition Control, National Engineering Research Center for Breeding Swine Industry, College of Animal Science, South China Agricultural University, Guangzhou, China; ^2^National Engineering Laboratory for Pollution Control and Waste Utilization in Livestock and Poultry Production, Key Laboratory of Agro-ecological Processes in Subtropical Region, Institute of Subtropical Agriculture, Chinese Academy of Sciences, Changsha, China

**Keywords:** polyphenol, gallic acid, gut microbiome, immune response, gastrointestinal health

## Abstract

Gallic acid (GA) is a naturally occurring polyphenol compound present in fruits, vegetables, and herbal medicines. According to previous studies, GA has many biological properties, including antioxidant, anticancer, anti-inflammatory, and antimicrobial properties. GA and its derivatives have multiple industrial uses, such as food supplements or additives. Additionally, recent studies have shown that GA and its derivatives not only enhance gut microbiome (GM) activities, but also modulate immune responses. Thus, GA has great potential to facilitate natural defense against microbial infections and modulate the immune response. However, the exact mechanisms of GA acts on the GM and immune system remain unclear. In this review, first the physicochemical properties, bioavailability, absorption, and metabolism of GA are introduced, and then we summarize recent findings concerning its roles in gastrointestinal health. Furthermore, the present review attempts to explain how GA influences the GM and modulates the immune response to maintain intestinal health.

## Introduction

Gallic acid (GA), 3,4,5-trihydroxybenzoic acid, is a polyphenol compound (1) and has gradually won a considerable amount of attention because it is ubiquitous in fruits, vegetables, and herbal medicines, such as grapes (2–4), gallnuts (5, 6), pomegranates (7, 8), and tea leaves (9, 10). In 1786, Carl Wilhelm Scheele, a famous Swedish chemist, was the first to identify and isolate GA and pyrogallic acid from plants (11). Since then, reports on GA and its derivatives have gradually increased, which has increased awareness in the understanding of GA. In addition to the edible uses of GA and its ester derivatives as flavoring agents and preservatives in the food industry (12, 13), there are also various kinds of studies on their biological and pharmacological activities, including antioxidant (14, 15), antimicrobial (16, 17), anticancer (18, 19), anti-inflammatory (20, 21), gastroprotective (22–25), cardioprotective (26, 27), neuroprotective (28–30), and metabolic disease prevention activities (31–33). To date, however, virtually no published studies exist on the mechanisms of action of GA through the gut microbiome (GM) and immune response.

Therefore, in this review, we first cover the physicochemical properties, absorption, and metabolism of GA and then summarize recent findings concerning their roles in gastrointestinal diseases. Moreover, the current review tries to shed light on the regulatory mechanism of GA through modulation of the GM and immune response. Finally, we summarize our findings based on the obtained information and provide an outlook for further investigations. Relevant references and data for this review were derived from the *Web* of *Science* and PubMed databases, from which we chose the most relevant literatures that have investigated the effect of GA and its derivatives on the treatment or prevention of gastrointestinal diseases, especially focusing on the GM and immune response.

## Physicochemical Properties of GA

**Figure 1:**
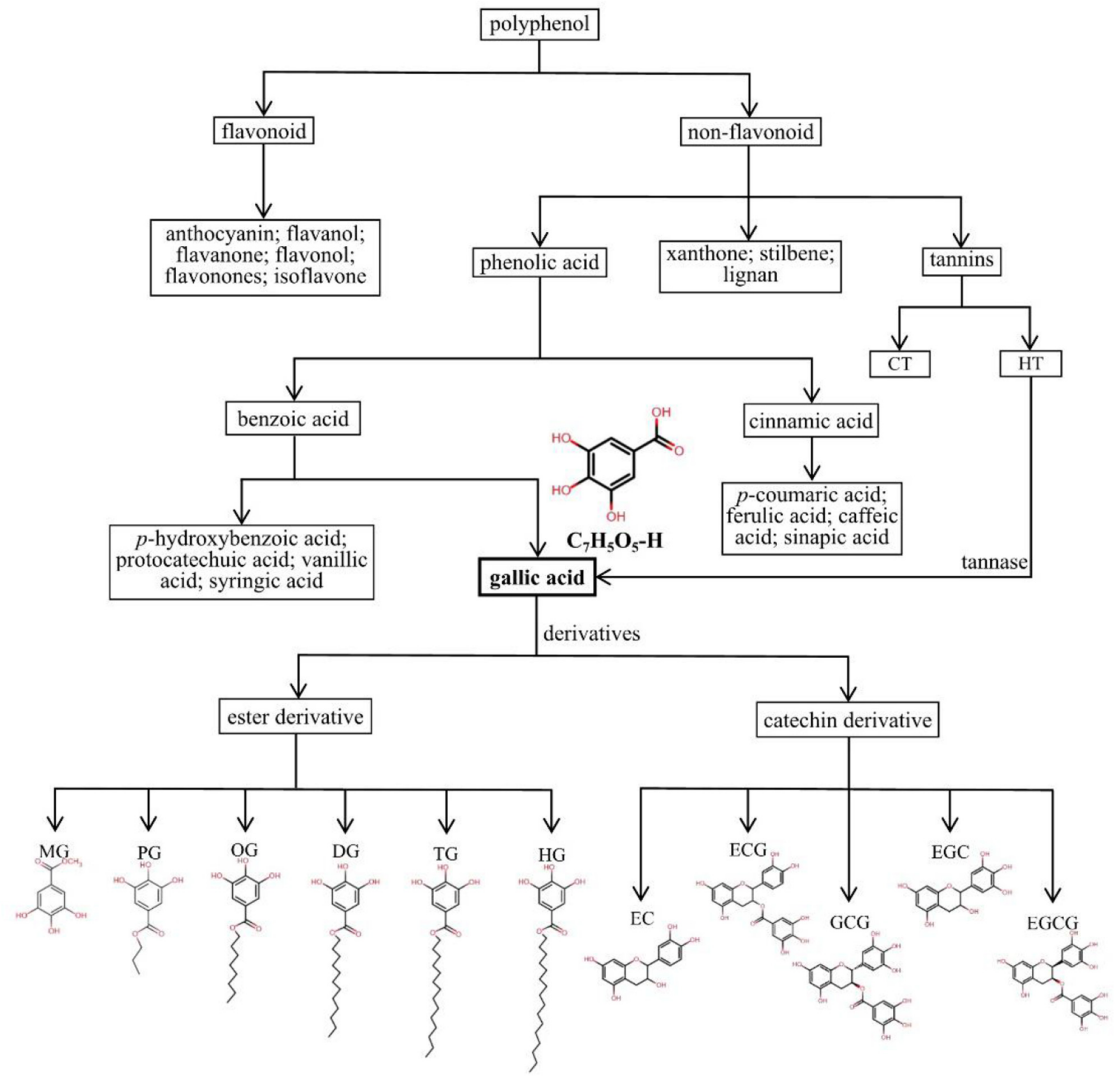
Detailed classification and chemical structures of polyphenols, phenolic acids, GA, and its derivatives. HTs, hydrolysable tannins; CTs, condensed tannins; MG, methyl gallate, C_7_H_5_O_5_-CH_3_; PG, propyl gallate, C_7_H_5_O_5_-(CH_2_)_2_-CH_3_; OG, octyl gallate, C_7_H_5_O_5_-(CH_2_)_7_-CH_3_; DG, dodecyl gallate, C_7_H_5_O_5_-(CH_2_)_11_-CH_3_; TG, tetradecyl gallate, C_7_H_5_O_5_-(CH_2_)_13_-CH_3_; HG, hexadecyl gallate, C_7_H_5_O_5_-(CH_2_)_15_-CH_3_; EC, epicatechin, C_15_H_14_O_6_; ECG, epicatechin gallate, C_22_H_18_O_10_; GCG, gallocatechin gallate, C_22_H_18_O_11_; EGC, epigallocatechin, C_15_H_14_O_7_; EGCG, epigallocatechin gallate, C_22_H_18_O_11_.

Frequently, polyphenols are mainly divided into two categories, including flavonoids (anthocyanins, flavanols, flavanones, flavonols, flavonones, and isoflavones) and non-flavonoids (phenolic acids, xanthones, stilbenes, lignans, and tannins). Phenolic acids arise from two major phenolic compounds: benzoic acids and cinnamic acids, separately based on the C1-C6 and C3-C6 backbones. *p*-Hydroxybenzoic acid, protocatechuic acid, vanillic acid, GA, and syringic acid are hydroxybenzoic derivatives, and hydroxycinnamic acids include *p*-coumaric acid, ferulic acid, caffeic acid, and sinapic acid (34, 35). Due to their different structures, hydroxycinnamic acids show higher antibacterial activity than hydroxybenzoic acids (36). Tannins are classified as hydrolysable tannins (HTs) and condensed tannins (CTs) (11). HTs contain a glucose unit and esterified gallic acid. As presented in Figure 1, GA is a trihydroxybenzoic acid with the molecular formula C_7_H_6_O_5_ and molecular weight of 170.12 g/mol, and hydroxy groups are at positions 3, 4, and 5. It is a colorless or slightly yellow crystalline compound, and the melting point is 210°C, with decomposition between 235°C and 240°C producing carbon dioxide and carbon monoxide. Its density is 1.69 kg/L, its pKa is 4.40, and its log *P* is 0.70 at 20°C. It is soluble in water, alcohol, ether, and glycerol, and practically insoluble in benzene, chloroform, and ether petroleum (1). GA is a secondary metabolite widely distributed in several fruits, vegetables, and herbal medicines (37), and it is used in photography, pharmaceuticals, and analytical reagents (38). GA is found both free and as part of HTs. It is the most basic constituent donor used to synthesize HTs through esterification of GA with glucose and products of their oxidative reactions. HTs contains mainly glucogallin, gallotannins, ellagitannins, and their derivatives (39). Tannase (a glycoprotein esterase) hydrolyzes GA from gallotannins, thereby increasing available GA absorbed in the gastrointestinal tract (GIT) (40, 41). The GA groups are usually bonded to form dimers, such as ellagic acid.

The GA derivatives include two types: ester and catechin derivatives. The most common ester derivatives of GA are alkyl esters, which are composed mainly of methyl gallate (MG), propyl gallate (PG), octyl gallate (OG), dodecyl gallate (DG), tetradecyl gallate (TG), and hexadecyl gallate (HG), and some of the main catechin derivatives are epicatechin (EC), epicatechin gallate (ECG), epigallocatechin (EGC), gallocatechin gallate (GCG), and epigallocatechin gallate (EGCG) (42–45). In particular, EGCG, a main bioactive compound, has been observed to have potent anticancer activities and protective effects on cardiovascular and metabolic diseases with multiple mechanisms (46–49). Owing to the properties of potent antioxidants scavenging of reactive oxygen species, several GA derivatives, such as DG, PG, OG, TG, and HG, are widely used in the food manufacturing, pharmaceutical, and cosmetic industries (43, 45, 50). The detailed classification and chemical structures of polyphenols, phenolic acids, GA and its derivatives are shown in Figure 1.

## Bioavailability, Absorption, and Metabolism of GA

It has been widely claimed that polyphenols are good source of natural health products and are beneficial for human health (51–55). Oliver et al. found that polyphenols have high instability to light, heat, and pH due to the existence of multiple hydroxyl groups (56). To a great extent, these external factors affect their commercial popularization and application. In addition, the poor solubility characteristics limit their wide application in the fields of food products and supplements (57, 58). Moreover, polyphenols are quickly absorbed in the GIT, with rapid metabolism within the human gut and a high elimination rate *in vivo*, resulting in low and inconsistent oral bioavailability (59–61). Similarly, as a phenolic acid in polyphenols, GA and its derivatives also have the above disadvantageous properties, poor bioavailability, stability, and solubility (3, 62). Fortunately, the developing colloidal delivery systems could significantly improve its bioavailability, which brings large possibility for application in human.

**Figure 2:**
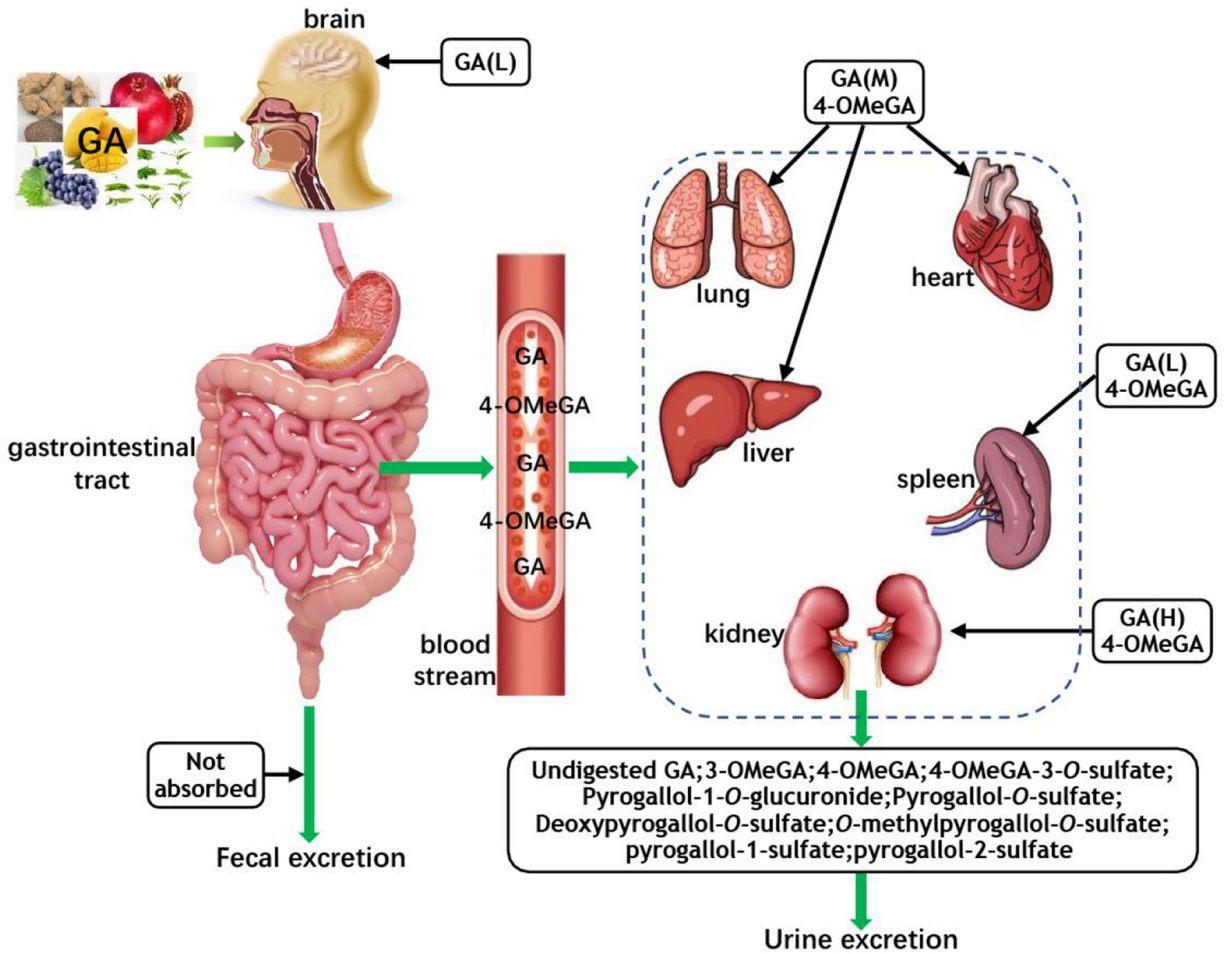
The absorption, metabolism, and distribution of GA. GA, gallic acid; 4-OMeGA, 4-*O*-methygallic acid; 3-OMeGA, 3-*O*-methygallic acid; H, M, and L represent the high, middle, and low content of GA in corresponding tissues and organs, respectively; the 4-OMeGA in black boxes represent that it is the primary metabolite of GA in different organs.

The 4-*O*-Methygallic acid (4-OMeGA) is the primary metabolite of GA in human plasma and urine (3, 63, 64). After oral administration, nearly 70% of GA is absorbed and then excreted via urine as 4-OMeGA (65, 66). Barnes et al. identified GA metabolites (pyrogallol-1-*O*-glucuronide, 4-OMeGA, 4-OMeGA-3-*O*-sulfate, pyrogallol-*O*-sulfate, deoxypyrogallol-*O*-sulfate, and *O*-methylpyrogallol-*O*-sulfate) in the urine of healthy volunteers over a 12 h period by tandem mass spectrometry (MS/MS) analysis after the consumption of 400 g/d Keitt mango for 10 days (67). Further study indicated that after a single oral administration of *Polygonum capitatum* extract at 60 mg/kg (equivalent to 12 mg/kg GA), GA was distributed mainly in rat kidney tissue (1,218.62 ng/g); the lung tissue had the second highest GA content (258.08 ng/g); the concentration of GA in the liver and heart was slightly lower than that of the lung; the spleen contained very little GA; and GA could not be found in brain tissue (62). However, a study suggested that the rat brain deposition of GA increased with repeated dosing of grape seed polyphenolic extract (3). In a urinary excretion study, approximately 16.67% of the intake GA was excreted in an unchanged form, and the predominant metabolite 4-OMeGA of GA was detected in the urine sample (62). The theaflavin galloyl moiety of black tea was consumed by GM, and the released GA was further transformed to 3-*O*-methyl GA (3-OMeGA), 4-OMeGA, pyrogallol-1-sulfate, and pyrogallol-2-sulfate, which were excreted via urine amounts to 94% of the intake (68). Figure 2 shows the absorption, metabolism, and distribution of GA *in vivo*.

These research results indicate that GA undergoes extensive metabolism after digestion, but its effectiveness is limited because of rapid metabolism and elimination. How to improve the bioavailability of GA remains a problem. To overcome these challenges, colloidal delivery systems have been tested to increase the intestinal absorption of GA and subsequently enhance its bioavailability in corresponding target organs and tissues. Natural proteins, polysaccharides, and biopolymer-based delivery systems have been widely used in the research of polyphenols (69), and colloidal encapsulation could enhance the efficacy of polyphenols in the field of food and biomedical applications (70, 71). Similarly, the phospholipid complexation delivery system also shows a good effect on GA. GA–phospholipid complex improved the bioavailability of GA by increasing absorption, decreasing elimination rate, and lengthening duration of action in rat serum (72). GA liposomes decorated with lactoferrin (LF-GA-LIP) could be developed as a favorable delivery system because it displayed a delayed-release effect in simulated digestion (73). Recent studies reported that the addition of tannase could enhance GA bioaccessibility in green tea and mango juice (40, 41). Repeated dosing and the use of structural analogs or derivative compounds of GA also were effective measures to improve the plasma levels of GA (3).

However, the application of these colloidal delivery systems still has many difficulties, such as the astringent taste of GA, and the bioavailability and potential toxicity of GA complexations should be explored through *in vitro* and *in vivo* trials. A more comprehensive understanding of GA is necessary. Only then can GA complexations be used safely and reasonably as supplements and drugs in production.

## GA in Gastrointestinal Health and Disease

Over the past decade, researchers have provided plenty of emerging evidence that the GM plays a crucial role in the maintenance of physiological homeostasis within the GIT, and microbiome dysbiosis is directly related to many health problems, such as gastrointestinal disease. Several studies in animal models investigate the effects of GA consumption on gastrointestinal diseases and its mechanisms of action.

### Gastric Cancer

Gastric cancer (GC) is one of the main causes of cancer deaths in the world and over 1 million new cases were diagnosed in 2018 (74). Gastric microbiota plays an initial role in GC (75), and infection with *Helicobacter pylori* (*H. pylori*) is the strongest risk factor linked to GC (76). Almost all cases of GC can be related to *H. pylori* (77). An aqueous extract of ginger (GA and cinnamic acid) could protect the gastric mucosa against stress induced mucosal lesions by suppressing *H. pylori*, blocking H^+^, K^+^-ATPase action, and providing antioxidant protection (25). The study suggests that GA has potential for prevention and treatment of GC through decreasing *H. pylori*; however, the aqueous extract was a mixture instead of pure GA; thus, the mechanism of action is uncertain, and further research is needed.

Meanwhile, GA has potent therapeutic effects on the non-steroidal anti-inflammatory drug (NSAID)-induced gastric mucosal damage by preventing oxidative stress and inhibiting the activation of the mitochondrial pathway of apoptosis in gastric mucosal cells (78). Similarly, gastric adenocarcinoma cell metastasis was inhibited by GA, whose possible mechanism may occur through inhibitory effects on the Ras/PI3K/AKT signaling pathway and transcriptional factor NF-κB, resulting in the antimetastatic effects (79). A study verified the protective mechanism of GA and its novel derivative [(E)-3,4,5-trihydroxy-N-(2-(piperazin-1-yl) ethyl) benzimidic acid] against ethanol-induced gastric ulcerogenesis, suggesting that the gastroprotective activity may be related to antioxidant properties, immunomodulatory markers, Hsp70 and Bcl-2-associated X protein, and inhibition of mitochondrial apoptosis (80). Interestingly, the combination of GA plus famotidine exhibited a synergistic role in the protection of rat gastric mucosa (81). This study provides a possibility for GA to enhance the therapeutic effect of antibiotics.

### Colorectal Cancer

Colorectal cancer (CRC) has the third highest cancer incidence around the world, and it constitutes a major global health burden threatening public health (82). The high number of studies have found that the GM plays a crucial role in colorectal carcinogenesis (83). Previous studies reported that dietary polyphenols benefit colorectal tissue integrity and function, gut bacterial growth and activities (84, 85). Although there is no direct evidence to suggest that GA prevents the occurrence of CRC by changing GM, we can indirectly speculate that GA influence the GM of CRC based on the related literatures of polyphenols and CRC.

GA and its derivative 3-OMeGA decreased human colon cancer cell viability by suppressing cell proliferation and regulating the signaling pathways of NF-κB, AP-1, STAT-1, and OCT-1 (86). Additionally, polymer nanoparticles assembled from GA-grafted chitosan (GA-g-CS) and caseinophosphopeptides (CPPs) were developed to deliver (-)-EGCG as novel functional foods. The GA-g-CS-CPP nanoparticles demonstrated powerful antioxidant activity and cytotoxicity against Caco-2 colon cancer cells, and the EGCG-loaded GA-g-CS-CPP nanoparticles further amplified the anticancer activity against Caco-2 cells (87). Similarly, GA-conjugated chitosan efficiently inhibited pulmonary metastasis of CT26 mouse colorectal carcinoma cells (88). In 1,2-dimethyl hydrazine-induced colon carcinogenesis in rats, the activity of phase II enzymes decreases, and phase I enzymes increases, whereas it is interesting to note that GA treatment could shift the above changes toward normal levels (89).

### Inflammatory Bowel Disease

Inflammatory bowel disease (IBD), including Crohn's disease (CD) and ulcerative colitis (UC), has long been doubted to correlate of an abnormal host reaction to GM (90). Both diseases are chronic and inflammatory disorders in the GIT with an increasing incidence rate being related to the rapid development of industrialization (91). Patients with these disorders have greater incidence to evolve into colon cancer (92, 93). Numerous experimental and clinical studies have indicated that various dietary polyphenols have beneficial effects against IBD (94–96).

GA could inhibit inflammation in dextran sulfate sodium (DSS)-induced colitis in mice through the suppression of p65-NF-κB and IL-6/p-STAT3Y705 activation (22), and suppress lipopolysaccharide (LPS)-induced inflammation in transgenic mice by regulating immune system processes and downregulating the NF-κB pathway (97). Li et al. was the first to perform *16S* gene sequencing on mice fecal and combined with metabolomics analysis; the results indicated that GA significantly attenuated UC by influencing composition of mice GM and metabolites (98). What makes us delighted is that a pilot study in patients with IBD found mango pulp (gallotannins and GA) intake markedly increased the abundance of beneficial bacteria such as *Lactobacillus spp., Lactobacillus plantarum, Lactobacillus reuteri*, and *Lactobacillus lactis*, which was accompanied by increased fecal butyric acid production (99).

However, there is only very limited evidence on the effectiveness of GA in GIT health, and very few human studies have been conducted on the impact of GA on GIT health. There is no adequate evidence to confirm the impact of GA on GIT health and disease. Further high-quality researches are needed to establish the mechanism of action of GA and its derivatives on GIT health. Several human studies have preliminarily interpreted the link between the GM and IBD. Thus, the GM could be a research direction between GA and GIT health in the future.

## Effects of GA on the GM

The GM is a key modulator of human health (100, 101). Trillions of microbes living in GIT finely regulate homeostasis in GIT ecosystem, most of which are beneficial to human health, affecting maintenance of the metabolic function of the host, development of the innate and adaptive immune systems, and resistance against invasion of enteric pathogens (102). In recent years, it has become a popular research hotspot in biomedical research because researchers have identified relationships between GM compositions and health (103). The microbial diversity and homeostatic configuration of the GM are affected by various factors, and diet appears to exert the greatest influence on the GM. Dietary components are utilized by the GM to produce energy and metabolites, which can mostly enter the bloodstream to affect intestinal function and the immune system (104). As an active ingredient in dietary polyphenols, GA has potent antimicrobial properties and is beneficial to human and animal health.

### Antimicrobial Properties *in vitro*

GA has broad-spectrum therapeutic properties including antibacteria, antifungal, and antiviral activities *in vitro* (Table 1). An *in vitro* study reported that GA suppressed viable bacteria and *Escherichia coli* (*E. coli*) biofilm formation by regulating *pgaABCD* gene expression (105); meanwhile, GA effectively inhibited *Shigella flexneri* biofilm formation and activity by regulating the expression of the *mdoH* gene and the *OpgH* protein (106), and had a specific antibiofilm effect on *Staphylococcus aureus* (*S. aureus*) by regulating the expression of the ica operon (107). Additionally, GA not only has potent anti-bacteria activity, but also against *Eumycetes* (36, 119). A storage test performed on fresh black truffles revealed the antimicrobial activity of GA observed *in vitro*, with a dramatic decline in the abundances of not only *Pseudomonas spp*., but also *Enterobacteriaceae* and *Eumycetes* (108), and it was observed that GA has a broad-spectrum antifungal activity for all tested dermatophyte strains (*Trichophyton rubrum, Trichophyton mentagrophytes, Trichophyton violaceum, Microsporum canis, Trichophyton verrucosum, and Trichophyton schoenleinii*) and Candida strains (*Candida glabrata, C. albicans, and Candida tropicalis*) (109). In addition, GA might be a sensitive reagent inhibiting influenza A (*H1N1*) virus infection (110) and has anti-HBV activity (111). Based on its powerful antimicrobial activity, GA is used to synthesize a kind of antimicrobial agent, such as trimethoprim, to treat some microbial infectious diseases (120).

**Table 1:** The antimicrobial activity of GA observed *in vitro*.

**Form**	**MIC/MBC**	**Change of strain**	**References**
GA	MIC in biofilm: 2 mg/mL; Minimal biofilm eradication concentration: 8 mg/mL	Inhibited *E. coli* biofilm formation by regulating *pgaABCD* genes expression	(105)
GA	MIC: 2 mg/mL; MBC: 8 mg/mL	Inhibited *Shigella flexneri* biofilm formation by regulating the expression of the *mdoH* gene and the *OpgH* protein	(106)
GA	MIC in suspension and in biofilms was 2 and 4 mg/mL	Inhibited *S. aureus* biofilm formation by regulating the expression of the ica operon	(107)
GA	MIC: 2.5 mg/mL; MBC: 10 mg/mL	Reduced the activity of *Pseudomonas spp., Enterobacteriaceae*, and *Eumycetes*	(108)
GA	MIC for dermatophyte strains: 43.75 ~ 83.33 mu g/mL MIC for Candida strains: 12.5~100.0 mu g/mL	Antifungal activity for dermatophyte strains (*T. rubrum, Trichophyton mentagrophytes, Trichophyton violaceum, Microsporum canis, Trichophyton verrucosum, Trichophyton schoenleinii*) and Candida strains (*Candida glabrata, C. albicans, Candida tropicalis*)	(109)
GA	The 50% effective inhibition concentration (EC50): 2.6 mu g/mL; The 50% cytotoxic concentrations (CC50): 22.1 mu g/mL	Inhibited influenza A (H1N1) virus infection	(110)
GA	7.01 mu g/mg	anti-HBV	(111)
GA + octyl gallate	MIC for GA: 3,150 mu g/mL; MIC for octyl gallate: 30 mu g/mL	Enhanced the inhibition of *Enterococcus faecalis* compared with the efficacy of individual compounds	(112)
Laccase-catalyzed chitosan–GA derivative	MIC for *S. aureus*: 0.2 mg/mL; MIC for *E. coli*: 0.4 mg/mL	Inhibited the growth of *E. coli* and *S. aureus*	(113)
GC-AgNps	MIC: 1 mu g/mL	Exhibited good antibacterial activity against *E. coli*	(114)
LF-GA-LIP	–	Exerted higher antibacterial properties against *E. coli* and *S. aureus* than GA-LIP	(73)
GA-g-chitin-glucan complex	–	Showed better antibacterial activity in comparison to unmodified chitin-glucan complex	(115)
GAGO	50–500 mu g/mL	Had potential anti-bacterial against *S. aureus*	(116)
Functionalized ZnO nanoparticles with GA	–	Displayed good antibacterial activity against methicillin-resistant *S. aureus* and *E. coli* compared to non-functionalized ZnO nanoparticles	(117)
GA and its derivatives (octyl gallate, propyl gallate)	–	The octyl gallate and propyl gallate had significant inhibition against Carnobacterium divergens ATCC 35677 and Leuconostoc carnosum ATCC 49367 originating from meat in comparison to GA	(17)
GA esters	MIC: 0.015 mg/mL	The 3-chloropropyl 3, 4, 5-trihydroxybenzoate against resistant gram-negative strains such as *P. aeruginosa, E. coli* and *E. aerogenes*	(118)

*MIC, minimum inhibitory concentration; MBC, minimal bactericidal concentration; GA, gallic acid; GC-AgNps, GA-chitosan-modified silver nanoparticles; LF-GA-LIP, GA liposomes decorated with lactoferrin; GA-g-chitin-glucan complex, GA-grafted chitin-glucan complex; GAGO, GA-loaded graphene oxide-based nanoformulation*.

The synergistic effects of natural antimicrobial compounds could increase the antimicrobial potential. A combination of GA and octyl gallate enhanced the antimicrobial activity for *Enterococcus faecalis* compared with the efficacy of individual compounds (112). A laccase-catalyzed chitosan–GA derivative markedly suppressed the growth of *E. coli* and *S. aureus*, and it could disrupt their cell membranes causing leakage of cytoplasm and increasing relative conductivity. Further, the cytotoxicity was notably decreased by proper modification of chitosan with GA (113). Furthermore, synthesized GA-chitosan-modified silver nanoparticles (GC-AgNps) exhibited good antibacterial activity against *E. coli* (114). LF-GA-LIP also exerted greater antibacterial capabilities against *E. coli* and *S. aureus* than GA-LIP (73). A GA-grafted chitin-glucan complex (GA-g-chitin-glucan complex) showed better antibacterial activity than the unmodified chitin-glucan complex (115). Shamsi et al. reported that a GA-loaded graphene oxide-based nanoformulation (GAGO) could be used as a potential antibacterial agent against *S. aureus* (116). The ZnO nanoparticles functionalized with GA displayed stronger antibacterial activity against methicillin-resistant *S. aureus* and *E. coli* compared with non-functionalized ZnO nanoparticles (117). Similarly, a study on chickens displayed a synergistic effect of GA and eugenol in reducing the heat lethality of *Salmonella spp*. (121).

GA and its derivatives (octyl gallate, propyl gallate) as well as binary combinations exhibit significant inhibition against *Carnobacterium divergens* ATCC 35677 and *Leuconostoc carnosum* ATCC 49367 originating from meat, and octyl gallate and propyl gallate were more effective than GA (17). Halogenated GA analogs might be promising drugs. Sherin et al. synthesized fifteen novel GA esters, and the most effective compound found was 3-chloropropyl 3,4,5-trihydroxybenzoate, a debenzylation of gallic acid ester, specifically against resistant gram-negative strains, such as *P. aeruginosa, E. coli* and *E. aerogenes* (118).

Such meaningful observations *in vitro* indicate that GA and its derivatives have antimicrobial activities, which can be strengthened by a favorable delivery system. However, *in vitro* studies raise a question of whether GA exerts healthy effects by changing the GM composition *in vivo*. Thus, studies *in vivo* in animals and humans need to be carried out.

### Action of GA on the GM in Animals and Humans

**Figure 3:**
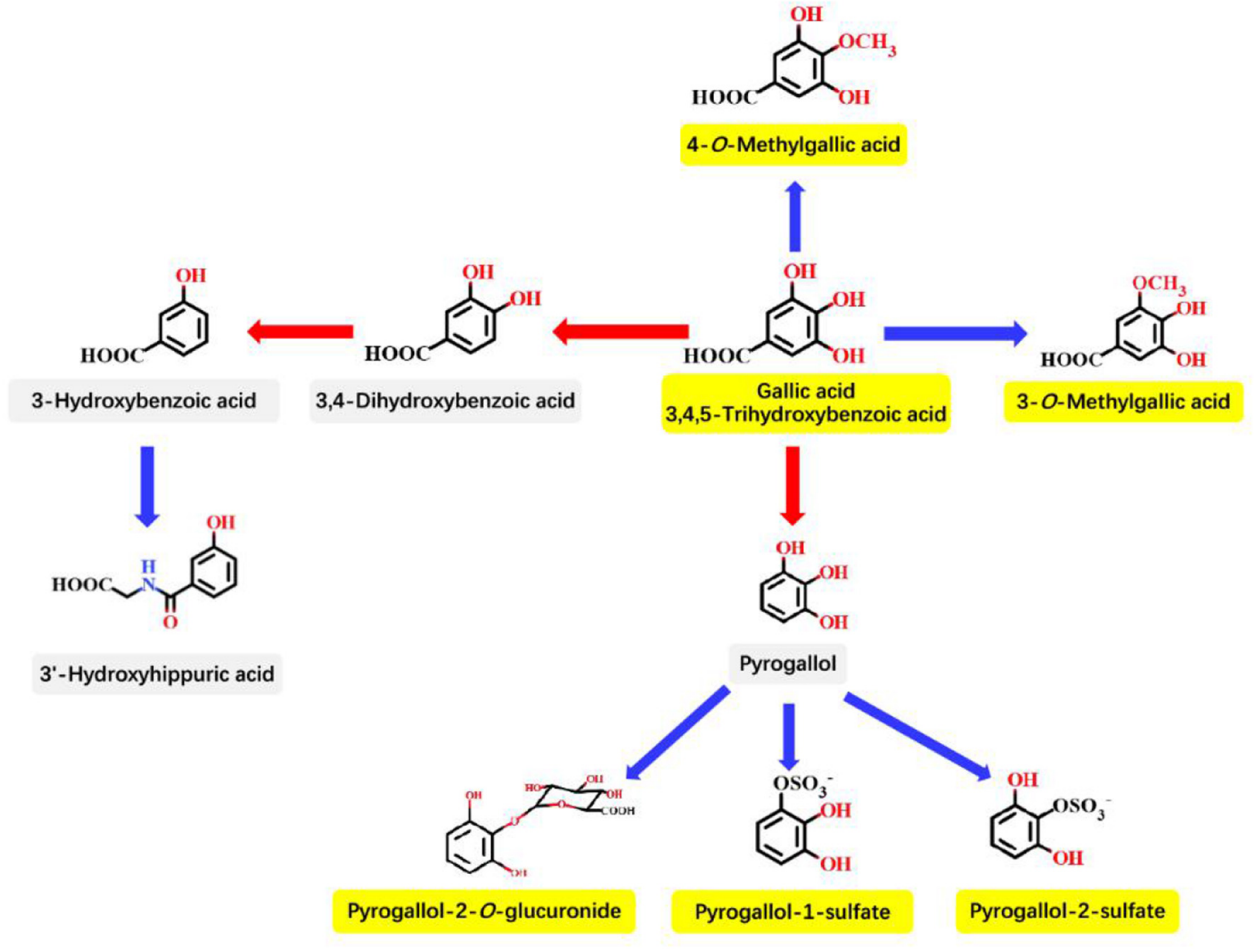
Proposed principle pathways for GA in the colonic microbiota and mammalian phase II metabolism. Red arrows indicate microbiota-mediated steps, and blue arrows represent mammalian enzyme-mediated conversions. The names in yellow boxes indicate the main metabolites accumulating in urine after GA intake. This figure is quoted from Pereira-Caro et al. (68).

Most plant-derived polyphenols must be transformed through the GM and intestinal enterocyte enzymes to be absorbed at enterocyte and colonocyte levels. The GM could transform polyphenols to final bioactive derivatives exhibiting antimicrobial properties. Therefore, an appropriate GM is extremely important for fighting against infectious diseases (122). Similarly, metabolism of GA in the GIT also requires the participation of the GM and intestinal enterocyte enzymes. As described in Figure 3 by Pereira-Caro et al. the principle pathways for GA in the colonic microbiota and mammalian phase II metabolism are proposed (68). The effect between the GM and GA is mutual; intestinal bacteria has the ability to metabolize GA, and GA also can induce changes in the microbiota toward a more favorable composition and activity, including the production of short-chain fatty acids (SCFAs) in the colon (22).

From the GM point of view, A recent study of animal model on the attenuation of DSS-induced rat UC by GA showed that GA dramatically decreased the GM abundance but had little impact on diversity. Changes in microbiota induced by DSS were characterized by a decrease in some probiotics predominantly in the *Lactobacillaceae* and *Prevotellaceae* families, and an increase in some pathogenic bacteria dominated by the *Firmicutes* and *Proteobacteria* phyla; GA reversed the above-mentioned changes and make them similar to the control group (22). Because fecal metabolites are byproducts of the interplay between the host and microbiota, changes in metabolites *in vivo* can act as evidence for alteration of the microbiota. Detection of the fecal SCFAs by gas chromatography concluded that the SCFAs contents were higher in the control group than GA and DSS group. Analysis of nuclear magnetic resonance-identified metabolites further revealed GA-induced metabolites changes mainly in increasing carbohydrate metabolism and bile acid metabolism and decreasing amino acid metabolism (22). All of above results demonstrate that GA-induced alterations in metabolites and GM in DSS-colitis provide new insight into the attenuation of UC by GA. Metabolomics data of rat plasma, liver, urine, and feces were analyzed by nuclear magnetic resonance whose results showed that changes in metabolites correlates to GA intake, and GA effectively promoted glycogenolysis, glycolysis, and TCA cycle and had positive effects on the metabolism of nucleotides, choline, bile acids, and amino acids (123). Fecal propionate and butyrate are fermentation products of insoluble polysaccharides and proteins (124). Remarkable increases in the levels of fecal propionate and butyrate and decreases in the levels of pyruvate, 2-ketoglutarate, lysine, alanine, and keto-acids suggested that GA could promote the GM fermentation of both proteins and polysaccharides. Research evidence suggests that GA has a great potential to be a natural antifungal agent for clinical application. A study in mice proved that intraperitoneal injection of GA markedly improved the rate of curability in a mouse model of systemic fungal infection (109).

In summary, available results *in vitro* and limited animal researches *in vivo* show GA can positively affect the composition of the GM or suppress the growth of pathogenic bacteria. However, it is a great pity that studies on the effects of GA and its derivatives on the human GM are lacking. The analysis of metabolites levels in human feces, urine, and blood combined with metagenomic analysis could offer a in-depth understanding of the impact of GA on humans.

## GA in Immunomodulation

The gut is an immune organ in which more than half of all immune cells are concentrated. The gut immune system linked to obesity, diabetes, food allergies, and IBD (125), thus, the gut immune function is closely related to human health. Various factors affect the development of the gut immune system, especially the GM and antigens, and they can drive the maintenance of gut barrier function and the development of the mucosal immune system (126). The mucus layer serves as the first protective barrier of the gut composed of an outer, loosely adhered layer and an inner, denser layer adhered to the underlying epithelium; the outer mucus layer is generally related to the GM (127). Immune dysfunction in the intestinal mucosa increases the risk of diarrhea in the host and has a negative impact on the balance of the GM (128), which could result in many serious consequences. Many studies have confirmed the regulatory role of plant-derived polyphenols in gut immune function (129, 130), thus fruits and vegetables rich in polyphenols are considered to be a preventive agent to promote intestinal health via modulating the intestinal mucosal immune response (127, 131).

The majority of immune-related disorders, such as pathogen-mediated infectious diseases, allergic diseases, and cancers, linked to inflammation (132). In the allograft model, GA accelerated the differentiation of T cells, increased the number of Tregs and exerted an anti-inflammatory effect, so GA has potential to treat diseases caused by excessive activation of immune cells (20). GA could decrease the exacerbated response of the body against an infectious agent to enhance innate immune activation by reducing the anti-apoptotic role of LPS, blocking the induction of neutrophil extracellular traps and preventing the formation of free radicals induced by LPS (133).

GA exhibited a protective effect against oxidative stress-induced cellular injury in human lymphocytes through immunomodulatory, antioxidant, and cytoprotective properties (134) and provided effective prevention against complications relating to immunological and thrombo-regulatory mechanisms via reverting the ATP and ADP hydrolysis and adenosine deaminase activity in lymphocytes, and preventing the increase in nucleoside triphosphate diphosphohydrolase, and adenosine deaminase activities in platelets (135). Additionally, GA inhibited the production of reactive oxygen species and nitric oxide, proinflammatory cytokine release, and phagocytes-induced lymphocyte proliferation in human peripheral blood mononuclear cells (136). The synergistic effect of GA and asparaginase also improved the antiproliferative effect on lymphoblastic cells (137).

GA could improve immunomodulatory activity by increasing of phagocytic capability, lysosomal volume, nitrite release, and intracellular calcium (${\text{Ca}}_{\text{i}}^{2+}$) levels in macrophages (138) and downregulate the MAPK-linked phagocytic signaling pathway in mouse murine macrophages (139). The efflux transporters P-glycoprotein and multidrug resistance proteins might participate in the transport of GA, and paracellular transport appeared to be the major limiting factor for the uptake of GA in Caco-2 cell monolayers (140). Polysaccharide nanofibers improved GA and EGCG permeability by opening the tight junctions of human differentiated epithelial Caco-2 cell monolayers and inhibiting efflux transporters (141). Cotreatment with curcumin and GA normalized the circulatory pro-inflammatory, anti-inflammatory cytokines, chemokines, N-εCML, CRP, and HbA1c (142). In addition, The GA derivatives (G-4, G-7, G-9, G-10, G-12, and G-13) also exhibited immunomodulatory activity and had high binding affinities for the INFα-2, IL-6, and IL-4 receptors, among which G-7 has the greatest immunomodulatory activity (143).

**Figure 4:**
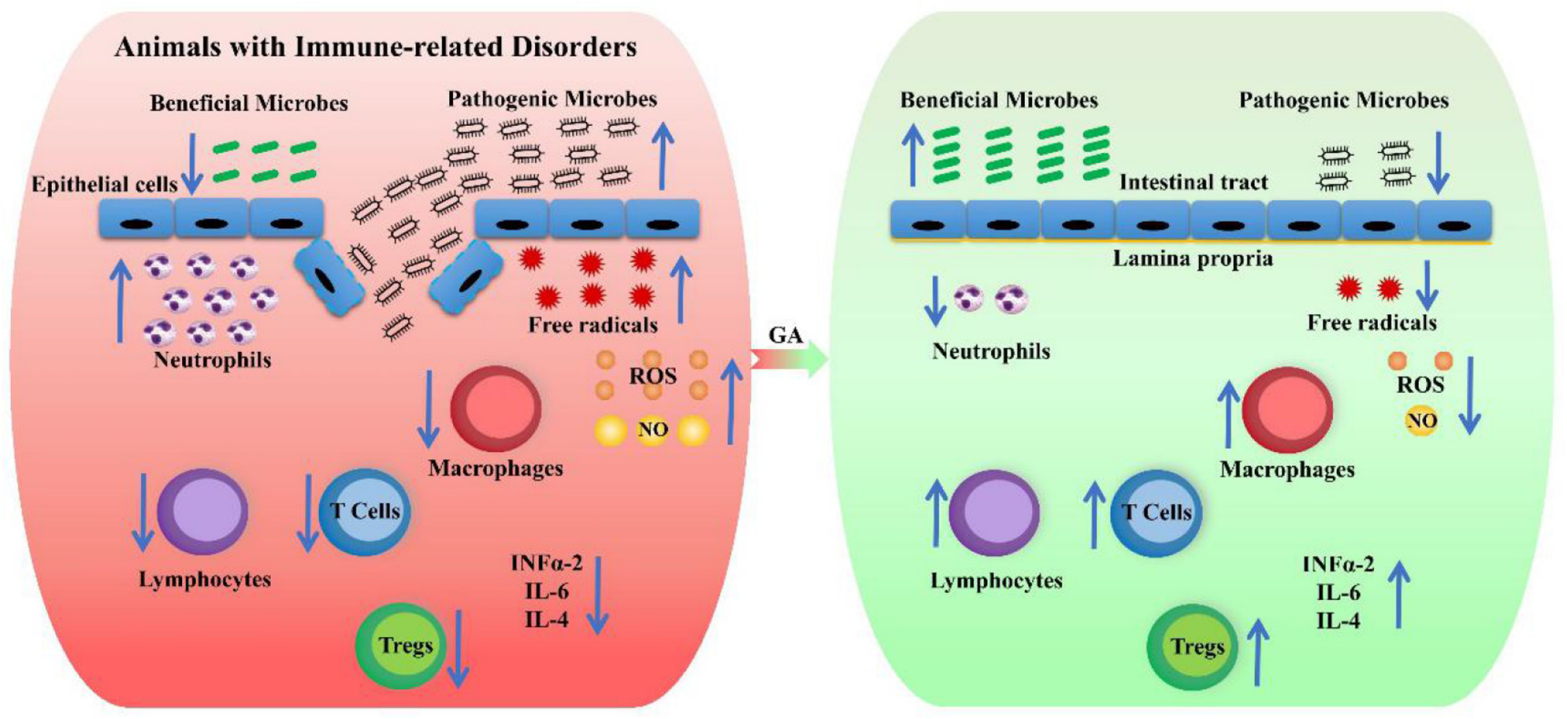
The possible mechanism of action of GA on the remission of immune-related diseases. The red box represents animals with immune-related disorders, and the green box indicates the attenuation effect of GA on immune-related disorders. The up arrows indicate a rising trend, and the down arrows show a declining trend.

The possible mechanism of action of GA on the remission of immune-related diseases is summarized in Figure 4.

The immunomodulatory activities of GA have not been deeply studied, and researchers should conduct a large number of experiments and preclinical studies on the immunomodulatory potential of GA to provide enough evidence to confirm the effectiveness and safety of GA. Furthermore, patients with immune-related diseases should be chosen as the research objects so that GA is further developed as a therapeutic agent for immune-related disorders.

## Conclusion and Outlook

This review summarizes the physicochemical properties and bioavailability of GA, and reports related to the impact of GA on gastrointestinal health focus mainly on GM, immunomodulation and mechanisms of action. According to these existing studies, GA and its derivatives have the potential to be novel agents for the treatment and prevention of gastrointestinal diseases through interaction with the GM and modulation of the immune response. Current *in vitro* evidence and results in animal models confirm the pharmacological and therapeutic interventions of GA. However, there is very limited clinical evidence for the effectiveness of GA in human gastrointestinal health and disease, and the exact underlying mechanisms of action are still obscure and unexplored. To clarify the interactions among the GM, immune response, and gastrointestinal disease in humans upon GA intervention, further investigation in other animal models and in humans is needed to verify the previous findings from animal models. Additionally, more efficient GA delivery systems need to be developed to improve GA bioavailability. With the rapid development of *omics* techniques, it is necessary and important to integrate genomics, transcriptomics, proteomics, and metabolomics to phenotyping to explore the molecular effects of GA in order to clarify its underlying mechanism of action.

## Author Contributions

KY generated ideas and wrote the initial manuscript. YY and BD guided and revised the manuscript. CT and JD made feasible suggestions for the manuscript. LZ, PL, ZXia, FZ, DS, and ZXin contributed to the collection and arrangement of literatures. All authors contributed to the article and approved the submitted version.

## Conflict of Interest

The authors declare that the research was conducted in the absence of any commercial or financial relationships that could be construed as a potential conflict of interest.
